# Unified framework for human and veterinary antibiotics: mapping international drug classifications (ATC and ATCvet) to support antimicrobial resistance research

**DOI:** 10.3389/fvets.2026.1796245

**Published:** 2026-05-07

**Authors:** Mélanie Daligault, François Caron, Albertine Leon, Julien Grosjean

**Affiliations:** 1 DYNAMICURE UMR 1311, Department of Digital Health, CHU Rouen, Univ Rouen Normandie, Rouen, France; 2 AIMS, Department of Digital Health, CHU Rouen, Univ Rouen Normandie, Rouen, France; 3 DYNAMICURE UMR 1311, Department of Infectious Diseases, CHU Rouen, Univ Rouen Normandie, Rouen, France; 4 Research Department, LABÉO, Saint-Contest, France; 5 DYNAMICURE UMR 1311, INSERM, UNICAEN, Normandie Univ, Caen, France

**Keywords:** antibiotic classification, antimicrobial resistance (AMR), ATC classification system, classification mapping, data interoperability, human antibiotics, veterinary antibiotics

## Abstract

Antimicrobial resistance (AMR) presents a global challenge requiring a One Health approach that integrates data from both human and veterinary sectors. However, cross-sector analyses are limited due to the lack of interoperability between antibiotic consumption and resistance datasets. The aim of the study was to map the Anatomical Therapeutic Chemical (ATC) classification used in human health and its veterinary equivalent, ATCvet. Systemic antibacterials from therapeutic groups J01 (ATC) and QJ01 and QJ51 (ATCvet) were selected for analysis. Automatic mapping was performed by pairing ATC and ATCvet codes based on corresponding codes. To illustrate the reuse of this reference framework, the criticality assessments from the World Health Organization (WHO) and the World Organisation for Animal Health (WOAH) were mapped onto this table, enabling automated combined analysis. The mapping identified 430 ATC + ATCvet codes, of which 59.3% showed strict equivalence between the two classifications. Additionally, there were 371 unique substances, with 73.0% found in both classifications. The majority of antibiotic classes were shared between the two classifications, while some such as pleuromutilins and quinoxalines were exclusive to veterinary medicine. The discrepancies in classification were primarily linked to specific characteristics of the ATCvet classification system and veterinary-specific indications. Integration with criticality assessments revealed broad correspondence between WHO and WOAH prioritization, with most critically important classes shared across sectors. The distribution of antibiotic classes across animal species showed extensive overlap, particularly within livestock species, underscoring the need for harmonized analyses. The ATC–ATCvet mapping provides a structured and interoperable framework suitable for cross-sector analyses of AMR. This harmonization enables consistent identification of antibiotic molecules, facilitates the integration of heterogeneous datasets, and supports One Health studies by bridging human and veterinary data sources.

## 1 Introduction

Combating antimicrobial resistance (AMR) requires a One Health approach, given the high consumption of antibiotics in both human and veterinary health (approximately 73% of global antimicrobial sales are attributed to livestock) and the cross-species transmission of resistance (1–3). While integrating data on antibiotic consumption and bacterial resistance is now manageable within each sector in many countries, combining them across sectors remains challenging due to a lack of interoperability. Indeed, some antibiotic molecules are reserved for specific animal species or for human use, often for reasons related to toxicity or legal regulations. However, even when the drugs are molecularly different, those within the same class may exert similar selective pressure on bacterial resistance. For example, third-generation cephalosporins (3GCs) are used in both human and veterinary medicine and can equally select for 3GC-resistant *Enterobacteriaceae*. The environment—specifically water, soil, and air—plays a significant role in the spread of both resistant bacteria and resistance genes, while anthropogenic activities increase the permeability of reservoirs (4). In this context, addressing antimicrobial resistance requires a multidisciplinary approach involving human, veterinary, and environmental health. However, implementing such a cross-sectoral approach may encounter challenges, particularly semantic ones, as the disciplines involved have traditionally operated within separate boundaries (5).

One effective strategy to improve interoperability and facilitate the analysis of species barrier crossing is to leverage classification systems that organize and classify concepts (6, 7). For substances used in human health, the Anatomical Therapeutic Chemical (ATC) classification, developed by the World Health Organization Collaborating Centre for Drug Statistics Methodology (WHOCC), serves as a reference, while its veterinary counterpart, ATCvet, extends this framework to animal health (8). The ATC classification system groups active medicinal substances according to the target organ or system and their therapeutic, pharmacological, and chemical properties. This classification is structured hierarchically, facilitating the identification of substances with the same target and properties, with each substance assigned to a unique code (9, 10).

This study aims to map antibiotic concepts from the ATC and ATCvet classifications and construct a shared reference table. This table could serve as a common foundation for querying databases relevant to AMR research in human and veterinary health, based on unified criteria. In addition, the enrichment of this dataset with supplementary references highlights key similarities and differences between the sectors, thereby supporting joint analyses.

## 2 Materials and methods

### 2.1 ATC and ATCvet classifications

The latest versions of both classifications were downloaded from official sources. For the ATC 2025 version, the French Digital Health Agency website provides a free version including English labels and their French equivalents, as the WHOCC allows ATC distribution at the national level. For the ATCvet classification, the original 26th edition was purchased from the Norwegian Institute of Public Health, which hosts the WHOCC (11–13). As the ATC and ATCvet classifications are based on the same coding system, the same substance with the same target corresponds to the same 7-character code, with the exception of the prefix Q used for all ATCvet codes. After removing the ATCvet prefix, the mapping process was conducted between the two classifications, automatically associating ATC codes with their strict ATCvet equivalents without considering labels. Codes specific to one classification, without an equivalent in the other, were also retained in the global dataset. From this dataset, systemic antibacterials used in human and veterinary medicine were selected from J01 (ATC) and QJ01 and QJ51 (ATCvet) therapeutic groups (ATC system level 2). For each antibiotic, higher-level classifications were preserved, with level 3 representing antibiotic families and level 4 representing antibiotic classes.

After case homogenization, a control step was performed to ensure that labels from the ATC and ATCvet classification systems corresponding to the same code were identical. This harmonization was applied across different ATC system code levels. In cases of minor discrepancies, labels were corrected to ensure consistency. For example, the ATCvet label “Pencillins” was changed to “Penicillins” or the phrase “for intramammary use” was removed. Any major discrepancies were identified. A second consistency check was performed to ensure that identical substance labels did not appear at different level-3 and level-4 positions within the ATC classification for systemic antibiotics between the ATC and ATCvet systems. As pharmacological and chemical characteristics should theoretically be consistent between the ATC and ATCvet systems, discrepancies between the two classifications were identified and reviewed. When differences in class definitions were observed, class labels were harmonized using the broadest denomination applicable to both classifications while retaining the original ATC and ATCvet codes. The objective of this procedure was to achieve a consistent and harmonized structure between the two classifications.

For antibiotic usage studies only, since the same antibiotic can be used in different medical contexts—including non-systemic use—and may be associated with different codes, all codes associated with the same antibiotic label were identified.

### 2.2 Application: enrichment with critical and species assessment

To illustrate the potential applications of this framework, we enriched the previously established foundation by integrating criticality assessments for systemic antibiotics from human and veterinary health organizations. For human health, the WHO’s critically important antimicrobial (CIA) list (2024) was used, and for veterinary health, the corresponding list from the 2024 World Organisation for Animal Health (WOAH) report was referenced. Both lists assign a level of criticality to antibiotics, structured by class and taking into account the lack of alternatives and the severity of infections. Following an automated harmonization of antibiotic labels (including case normalization and replacement of “+” or “−” with “and”), the lists were mapped onto the ATC + ATCvet reference using substance labels. For comparison purposes, the matching between the two criticality lists was also performed without relying on the ATC + ATCvet reference under the same conditions.

The WOAH VCIA list also includes animal species or families intended for food production associated with each substance’s use. In addition to the list of animal species, molecules listed by the WHO CIA were identified as intended for human use.

The analyses were carried out using R version 4.0.4 and the Tidyverse package version 2.0.0. The final dataset is available in the Supplementary Table S1.

## 3 Results

### 3.1 Antibiotic mapping

**Figure 1:**
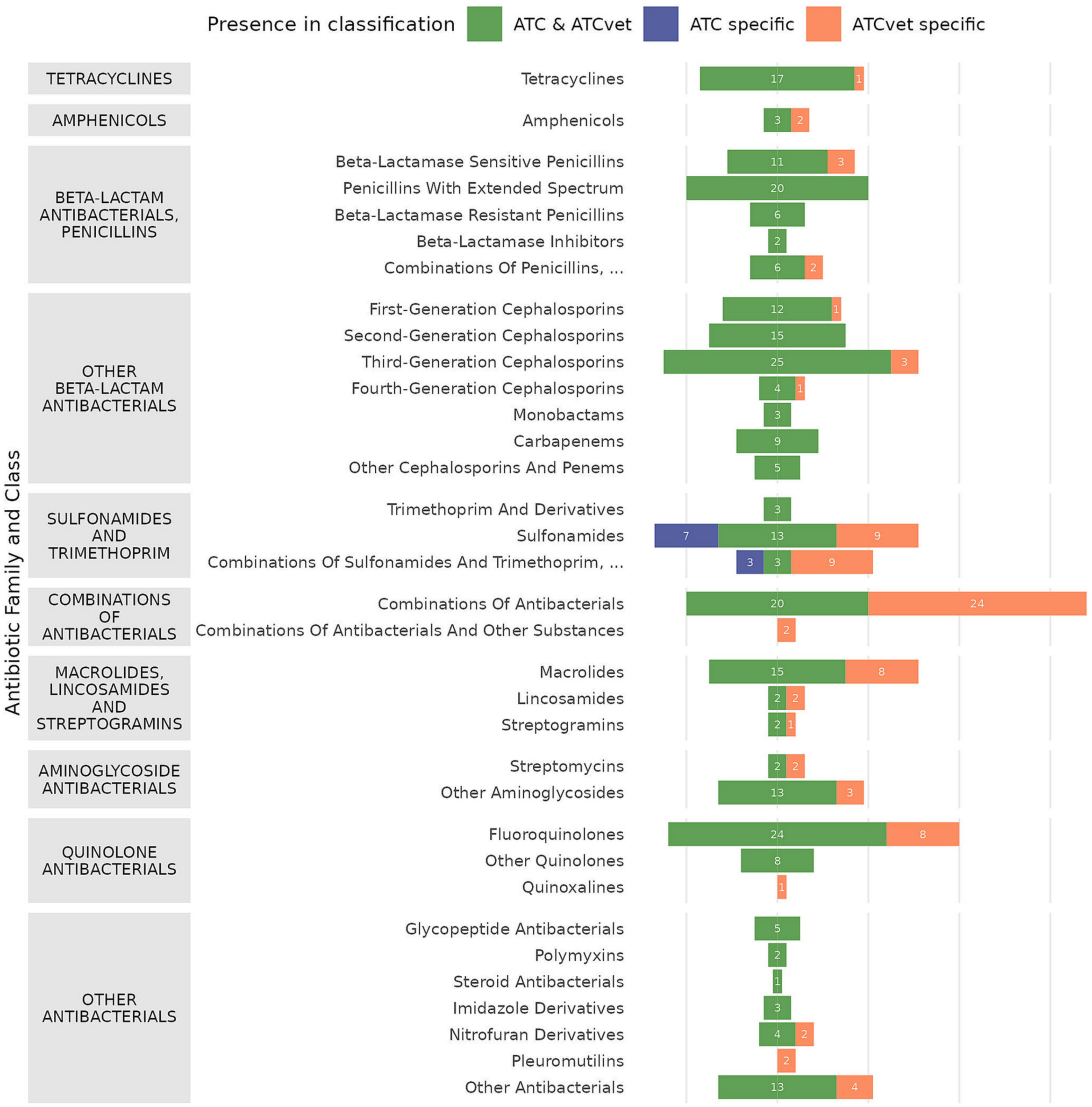
Distribution of antibiotic substances according to the ATC and ATCvet classifications, by antibiotic family and class (*n* = number of corresponding substances).

The selection process identified 285 ATC codes corresponding to 281 unique labels in human health and 400 ATCvet codes corresponding to 361 unique labels in veterinary health. The combined dataset comprised 430 ATC + ATCvet codes and 371 unique antibiotic substance labels. Mapping these classifications revealed 255 codes with direct ATC and ATCvet equivalents, accounting for 59.3% of the total selected codes. These codes were organized into 10 families and 34 antibiotic classes. The details of the mapping by code are provided in Supplementary Figure S1. All antibiotic families were represented in both ATC and ATCvet classifications. However, the pleuromutilins, quinoxalines, and “Combinations of antibacterials and other substances” classes were unique to the ATCvet classification and absent from the ATC classification (Figure 1). Among 371 unique chemical substance labels, 271 were shared between the ATC and ATCvet classifications for systemic use selection, 10 were exclusive to the ATC classification (all sulfonamides and trimethoprim), and 90 were specific to the ATCvet classification across different classes (Figure 1, Supplementary Figure S1).

### 3.2 Classification differences

The same antibiotic substance, used in different therapeutic contexts, such as systemic or ophthalmic use, can be assigned to multiple ATC system codes. Among the 371 substances identified, 219 exhibited exact one-to-one correspondence between their full ATC and ATCvet codes, reflecting potential use in the same type of indication in human and veterinary health. Of the remaining 152 substances, 92 were specific to one classification, including non-systemic antibiotics, while 60 substances had equivalents in both ATC and ATCvet classification systems but differed in their classification, reflecting differences in medical indications. For 43 of these substances, the differences were linked to ATCvet-specific codes. These included three ATCvet level-2 codes created specifically for veterinary use (QJ51 for intrauterine use, QG51 for intramammary use, and QP51 for antiprotozoal/antiparasitic agents) and one ATCvet level-4 group (QJ01EQ for sulfonamides, corresponding to ATC groups J01EB, J01EC, and J01ED), which were classified differently due to distinct pharmacokinetic properties in animals (10). The “combinations” substance label was excluded due to its non-specific nature, corresponding to 38 ATC codes and 44 ATCvet codes, not all of which represented antibiotic combinations. The remaining 16 substance labels showed usage differences between the ATC and ATCvet classification systems that were unrelated to ATCvet-specific classification (Table 1). Among these, metronidazole, ornidazole, and tinidazole from the “imidazole derivatives” class were classified as antiprotozoals (P01AB) in the ATC classification system only. The remaining 13 substances had additional indications in the ATCvet classification system. Sulfacetamide, combinations of sulfonamides, virginiamycin, dihydrostreptomycin, framycetin, paromomycin, furazolidone, and rifaximin were categorized as “Antibacterials for systemic use” (QJ01) in veterinary health but were not listed as such in the ATC classification system. These eight antibiotics were also part of the 90 antibiotics specific to the ATCvet classification system, which is true only for systemic use. Among the others, bacitracin is used as an intestinal anti-infective (QA07A), cloxacillin is used as an ophthalmic anti-infective (QS01AA), tetracycline is used as a gynecological anti-infective (QG01A) exclusively in the ATCvet classification, oxytetracycline combinations are used for dermatological use (QD06A), and gentamicin can be used both as an intestinal anti-infective (QA07A) and a gynecological anti-infective (QG01A) in veterinary health.

**Table 1:** Antibiotic substances shared between ATC and ATCvet classifications with differing system codes, reflecting differences in target organs or systems (*n* = number of listed codes).

Antibiotic class	Antibiotic label	ATC codes	ATCvet codes
Tetracyclines	tetracycline	*A01AB13*; *D06AA04*; J01AA07; *S01AA09*; *S02AA08*; *S03AA02* (*n* = 6)	*QA01AB13*; *QD06AA04*; ***QG01AA90***; ***QG51AA02*** ; QJ01AA07; **QJ51AA07** ; *QS01AA09*; *QS02AA08*; *QS03AA02* (*n* = 9)
	oxytetracycline, combinations	J01AA56 (*n* = 1)	***QD06AA53**;* QJ01AA56 (*n* = 2)
Beta-lactamase-resistant penicillins	cloxacillin	J01CF02 (*n* = 1)	QJ01CF02; **QJ51CF02** ; ***QS01AA90*** (*n* = 3)
Sulfonamides	sulfacetamide	*D10AF06*; *S01AB04* (*n* = 2)	*QD10AF06*; **QJ01EQ21** ; *QS01AB04* (*n* = 3)
	combinations of sulfonamides	*G01AE10 (n = 1)*	*QG01AE10*; **QJ01EQ30** ; ***QP51AG30*** (*n* = 3)
Streptogramins	virginiamycin	*D06AX10* (*n* = 1)	*QD06AX10*; **QJ01FG90** (*n* = 2)
Streptomycins	dihydrostreptomycin	*S01AA15* (*n* = 1)	***QA07AA90***; **QJ01GA90**; **QJ51GA90** ; *QS01AA15* (*n* = 4)
Other aminoglycosides	gentamicin	*D06AX07*; J01GB03; *S01AA11*; *S02AA14*; *S03AA06* (*n* = 5)	***QA07AA91***; *QD06AX07*; ***QG01AA91***; ***QG51AA04*** ; QJ01GB03; **QJ51GB03** ; *QS01AA11*; *QS02AA14*; *QS03AA06* (*n* = 9)
	framycetin	*D09AA01*; *R01AX08*; *S01AA07* (*n* = 3)	*QD09AA01*; **QJ01GB91**; *QR01AX08*; *QS01AA07* (*n* = 4)
	paromomycin	*A07AA06* (*n* = 1)	*QA07AA06*; **QJ01GB92** (*n* = 2)
Imidazole derivatives	metronidazole	*A01AB17*; *D06BX01*; *G01AF01*; J01XD01; ***P01AB01*** (*n* = 5)	*QA01AB17*; *QD06BX01*; *QG01AF01*; QJ01XD01; ***QP51CA01*** (*n* = 5)
	ornidazole	*G01AF06*; J01XD03; ***P01AB03*** (*n* = 3)	*QG01AF06*; QJ01XD03; ***QP51AA03*** (*n* = 3)
	tinidazole	*G01AF21*; J01XD02; ***P01AB02*** (*n* = 3)	*QG01AF21*; QJ01XD02; ***QP51AA02*** (*n* = 3)
Nitrofuran derivatives	furazolidone	*G01AX06* (*n* = 1)	*QG01AX06*; **QJ01XE90** (*n* = 2)
Other antibacterials	bacitracin	*D06AX05*; J01XX10; *R02AB04*; *S01AA32* (*n* = 4)	***QA07AA93***; *QD06AX05*; QJ01XX10; *QR02AB04*; *QS01AA32* (*n* = 5)
	rifaximin	*A07AA11*; *D06AX11* (*n* = 2)	*QA07AA11*; *QD06AX11*; ***QG51AA06*** ; **QJ51XX01** (*n* = 4)

**Bold**: Codes not shared between ATC and ATCvet; Underline: ATCvet-specific groups; *Italic*: Codes outside the initial scope.

### 3.3 Mapping criticality levels to the ATC + ATCvet classification

The WHO provides criticality assessments for 329 antibacterial substances, of which 244 could be mapped to the ATC + ATCvet classification, while the WOAH lists 127 substances, of which 87 could be mapped. Only 78 substances were shared across all three lists, accounting for 21% of the 371 previously identified substances. In addition, 109 antibiotics listed in at least one of the two criticality assessments were not present in the ATC + ATCvet classification. Moreover, three main factors may explain this discrepancy: (i) some substances are not classified as systemic antibiotics in the ATC system (e.g., rifampicin, classified as a treatment for tuberculosis), (ii) some substances are entirely absent from the ATC classification (e.g., semduramicin), and (iii) some substances are present in the ATC system but under a different label (e.g., “amoxicillin + clavulanic acid”, listed as “amoxicillin with a beta-lactamase inhibitor”).

The results of this mapping can also be examined at the level of antibiotic class groupings, which is more relevant for antimicrobial resistance monitoring. The CIA and VCIA lists both provide antibiotic groupings, comprising 55 and 40 groups, respectively. However, these groupings are not directly comparable between the two lists, as only 16 of the 94 shared molecules have identical group labels. In the remaining cases, the groupings are similar but not identical—for example, “Quinolones” versus “Quinolones (first generation)” or “Penicillins (narrow spectrum)” versus “Phenoxypenicillins.” The ATC + ATCvet system allows antibiotic classes defined in the classification to be inherited by all mapped substances, resulting in 30 classes represented in the CIA list and 22 in the VCIA list, of the 34 identified classes. Criticality can be assessed at the antibiotic class level, meaning that the evaluation of a single class member can determine thecriticality of the entire class. Most antibiotic classes fall within a single criticality category, with only a few exceptions.

The results of this characterization of criticality levels by antibiotic class are presented in Table 2. Antibiotic classes are not equally distributed across criticality levels, with a marked imbalance favoring critically important molecules, particularly in veterinary health (Table 2). Among the classes evaluated by the WHO and WOAH, fluoroquinolones, third- and fourth-generation cephalosporins, macrolides, streptomycin, and aminoglycosides are classified as critically important in both human and veterinary health. In contrast, no common class is ranked at the lowest criticality level “Important Antimicrobial.” Only four classes are classified at the lowest criticality level in veterinary health, including two not authorized for human use (novobiocin and olaquindox) and two classified as “Highly important antimicrobials” in human health. Meanwhile, in human health, four other classes are ranked at the lowest criticality level but are considered “Critically important” or “Highly important Antimicrobial Agents” in veterinary health. The intersection between “Veterinary Critically Important Antimicrobial Agents “and human “Highly Important Antimicrobials” represents the largest number of shared antibiotic substances.

**Table 2:** Comparison of WOAH and WHO criticality classifications for antibiotic classes (*n* = number of listed substances).

WHO CIA/WOAH VCIA	VCIA (*n* = 68)	VHIA (*n* = 15)	VIA (*n* = 4)	Not available (*n* = 166)
HPCIA (*n* = 62)	Fluoroquinolones Fourth-generation cephalosporins Third-generation cephalosporins	Other antibacterials: fosfomycin Other quinolones		Polymyxins
CIA (*n* = 38)	Macrolides Other aminoglycosides Streptomycins	Other antibacterials: rifaximin		
HIA (*n* = 101)	Amphenicols Beta-lactamase-resistant penicillins Beta-lactamase-sensitive penicillins Penicillins with extended spectrum Sulfonamides Tetracyclines Trimethoprim and derivatives	First-generation cephalosporins Lincosamides Second-generation cephalosporins	Steroid antibacterials Streptogramins	Combinations Of Penicillins, Incl. Beta-lactamase inhibitors Imidazole derivatives
IA (*n* = 9)	Other antibacterials: spectinomycin	Other antibacterials: bacitracin Pleuromutilins		Nitrofuran derivatives
Authorized for use in humans only (*n* = 32)	Penicillins with extended spectrum			Carbapenems Glycopeptide antibacterials Macrolides Monobactams Other aminoglycosides Other antibacterials: clofoctol; nitroxoline; linezolid; daptomycin; tedizolid Other cephalosporins and penems Tetracyclines
Not authorized for use in humans (*n* = 2)			Other antibacterials: novobiocin Quinoxalines	

HPCIA, highest priority critically important antimicrobial; CIA, critically important antimicrobial; HIA, highly important antimicrobial; IA, important antimicrobial; VCIA, veterinary critically important antimicrobial; VHIA, veterinary highly important antimicrobial; VIA, veterinary important antimicrobial.

### 3.4 Distribution by animal species

**Figure 2:**
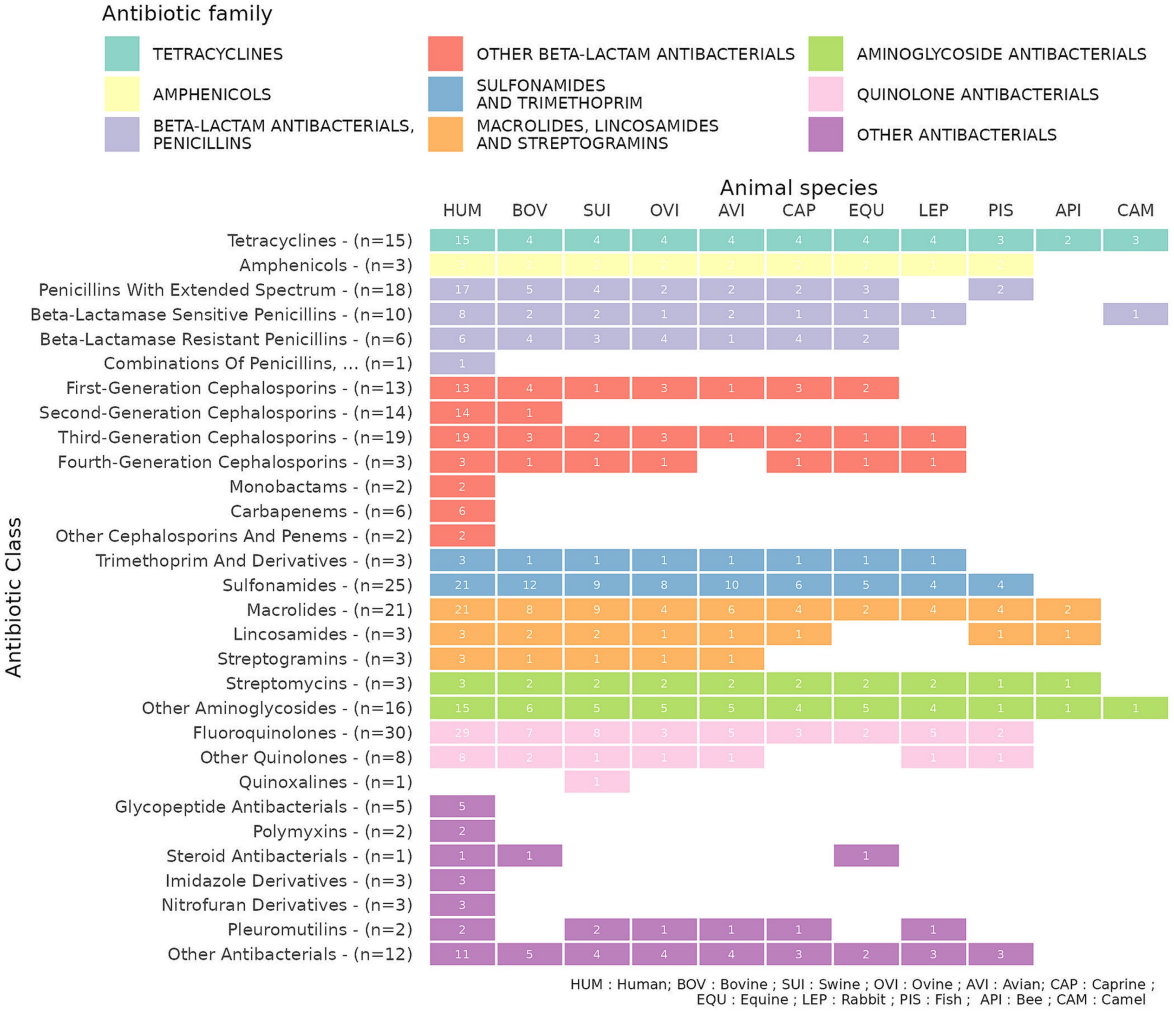
Distribution of ATC + ATCvet antibiotic substances by antibiotic class and animal species, according to the WHO and WOAH classifications (*n* = number of listed substances).

Among the 34 antibiotic classes identified in the ATC + ATCvet system, only 4—“Beta-lactamase inhibitors,” “Combinations of sulfonamides and trimethoprim, Incl. Derivatives,” *“*Combinations of antibacterials,” and “Combinations of antibacterials and other substances”—could not be assigned to animal species due to their absence from the WHO and WOAH criticality assessment lists. A significant disparity exists in the distribution of antibiotic classes across the animal species studied. The families of Tetracyclines and Aminoglycosides are classified as being used across all listed animal species. Amphenicols and Sulfonamides and Trimethoprim are used in a wide range of food-producing animal species, with the exception of bees and camelids. For some families, the range of species depends on the specific antibiotic class. For example, the Quinolone family covers a broad range of species; however, Quinoxalines are exclusively used in swine and represent the only antibiotic class without a human-use equivalent. Conversely, several classes are restricted to human health, including Monobactams, Carbapenems, Glycopeptides, and Imidazole and Nitrofuran derivatives. In total, 18 of the 30 antibiotic classes represented are present in more than half of the listed species, including most livestock species (Figure 2).

## 4 Discussion

The shared structure between the ATC and ATCvet classification systems enables highly coherent mapping of antibiotic molecules across human and veterinary health. This common framework serves as a valuable tool for jointly analyzing data sources, particularly those related to antibiotic consumption in both sectors. Some adjustments and additions have been made within the ATCvet classification system to reflect the specific uses or pharmacokinetic characteristics of several molecules (10). Therefore, selecting codes of interest based solely on ATC data may lead to the omission of specialized codes, which could be deleterious and result in the loss of a significant portion of the data. A primary example is the groups dedicated to intramammary use (QJ51 and QJ54), given that mastitis accounts for 40% of antibiotic administration for medical indication in cattle (14). In addition, because groupings may differ between classifications, directly transferring codes from one system to another without checking their completeness can be misleading, particularly for sulfonamides and combination therapies. This study provides support for selecting codes and for mapping ATC codes to ATCvet codes, as well as ATCvet codes to ATC codes. This approach may be relevant for the development of integrated surveillance programs, as encouraged by many organizations (15).

In addition to these classification differences, the mapping revealed a strong correspondence in the representation of antibiotics, with only 92 substances lacking equivalents between the two sectors, roughly one-third representing combinations, and 16 listed with differing uses. While these data do not indicate the level of use of each antibiotic across sectors, they confirm the strong overlap in the catalogue of antibiotic molecules and underscore the importance of coordinated use across sectors. Although the initial scope of systemic antibacterials is not exhaustive—as some antibiotics may be classified differently, such as systemic antifungals, antituberculosis agents, or non-systemic agents—this approach can be easily expanded to include additional antibiotic substances based on other defined criteria and can also be extended to anti-infective agents more generally.

Mapping criticality data onto the ATC + ATCvet system demonstrates the utility of a common framework for structuring such data, particularly by enabling the inheritance of characteristics from one data source to another. A key limitation is that mapping relies on antibiotic substance labels, which may vary across sources. Therefore, a standardization step is essential, although it cannot ensure perfect correspondence. The high similarity between certain molecule labels limits the applicability of approximate matching methods, such as fuzzy joins (e.g., ronidazole and ornidazole). In such cases, manual mapping guided by expert knowledge is required to ensure completeness. The use of standardized codes, as provided by the ATC system, overcomes nomenclature inconsistencies and facilitates data interoperability. This approach has been specifically chosen by the WHO AWaRE classification, the AMEG classification, and the ESAC-Net and ESUAvet initiatives, further enabling integration with other data resources (16–19). This methodology could be applied to additional data sources, especially antibiotic consumption data from both human and animal health sectors, to enable easier integration and analysis.

Nevertheless, the mapping of the two criticality assessment lists demonstrated a significant overlap in critical antibiotics for both human and veterinary health. This underscores the importance of monitoring antibiotic resistance, as the development of resistance in a critical antibiotic class in animals could have an impact on human health, regardless of the transmission pathway. These findings should also be considered in the context of local regulations that restrict the use of certain antibiotics in animals. For example, streptogramins (J01FG and QJ01FG) are present in both classifications; however, they are classified in the AMEG category A (‘Avoid’), which prohibits their use in food-producing animals and strongly restricts their use in companion animals in countries that rely on this classification to establish their regulatory framework (17, 20). In addition to regulations, there are also differences in practices regarding the use of antibiotics, both between human and veterinary healthcare and across different countries (21, 22).

Focusing on the animal species associated with different antibiotic classes reveals a substantial overlap in usage, with most classes represented across a broad range of animal species, particularly those commonly raised for meat production (poultry, cattle, goats, sheep, and pigs), as well as equines. This underscores the risk that the development of resistance could affect not only a wide array of animal species and their associated industries but also human health, as significant associations have been reported between veterinary antibiotic consumption and antimicrobial resistance in humans (23). The diminished efficacy of these antibiotics poses a threat to human and animal health, regardless of the source of antibiotic resistance. In this context of shared use of an arsenal between the two sectors, collaboration between them is essential to prevent antibiotic use in one sector from driving resistance in the other, particularly in animals intended for food production (24). In addition, companion animals, which are often prescribed the same antibiotic classes but under less stringent regulations than food-producing animals, may also play a non-negligible role in resistance dissemination (25, 26).

These results reflect only the number of substance codes and are not indicative of their level of use. For example, fluoroquinolones comprise the class with the highest number of molecules included in both the ATC and ATCvet classifications (24 and 32, respectively), yet they are not among the most widely consumed antibiotic classes in either human or veterinary medicine (23). This can be explained by the high degree of criticality assigned to this class in both sectors, which has led to restrictions on their use when alternatives are available (“Watch” in the WHO AWaRe classification and category B corresponding to “Restrict” in the AMEG classification) (16, 17, 27, 28).

The ATCvet classification has been integrated into the multi-terminology HeTOP server while preserving the existing links between ATC and ATCvet concepts (29). This also enables indirect connections between ATCvet concepts and those from other terminologies through the ATC classification, such as MeSH (Medical Subject Headings) descriptors used for bibliographic indexing or SNOMED CT (Systematized Nomenclature of Medicine Clinical Terms), which represents the most comprehensive medical nomenclature. This structuring and association of concepts facilitates data exploitation and enhances interoperability, which is also a major consideration for the development of artificial intelligence tools (30). This is particularly relevant given that integrated One Health surveillance is one of the four pillars of the global strategy to combat antimicrobial resistance (31).

## 5 Conclusion

In the fight against the threat of antimicrobial resistance, the ATC + ATCvet combination is a valuable tool for jointly analyzing data related to resistance and for establishing molecule references or classifications. Given its integration of therapeutic, chemical, and pharmacological concepts, this framework is particularly useful for the targeted identification of antibiotic molecules and for the stratification of data based on the same criteria used for antibiotic classes. However, due to variability in the labeling of antibiotic families, classes, and substances, careful review remains essential to accurately associate data from external sources with this classification. Widespread adoption of the ATC and ATCvet classifications offers a promising approach to improving data sharing and facilitating cross-disciplinary exchanges between human and veterinary health at the international level by relying on common criteria. Using this shared reference framework, this study highlights important overlaps in antibiotics regarding substances, indications, criticality, and animal species. Therefore, promoting data interoperability is essential to support a multidisciplinary approach and effectively implement One Health initiatives.

## Data Availability

The original contributions presented in the study are included in the article/Supplementary material, further inquiries can be directed to the corresponding author.

## Supplementary material

SUPPLEMENTARY FIGURE S1 Mapping of antibiotics for systemic use between the ATC and ATCvet classifications, represented in a Sankey diagram.

SUPPLEMENTARY TABLE S1 Results of the mapping of antibiotics for systemic use between the ATC and ATCvet classifications.
